# Examination of rapid adjustment system based on screen score obtained using continuous shear wave elastography

**DOI:** 10.1007/s10396-024-01439-7

**Published:** 2024-04-12

**Authors:** Marie Tabaru, Ren Koda, Hitoshi Shitara, Hirotaka Chikuda, Yoshiki Yamakoshi

**Affiliations:** 1 Institute of Innovative Research, Tokyo Institute of Technology, 4259 R2-25, Nagatsuta-cho, Midori-ku, Yokohama-shi, Kanagawa 226-8503 Japan; 2 Graduate School of Science and Technology, Gunma University, 1-5-1 Tenjin-cho, Kiryu-shi, Gunma 376-8515 Japan; 3 Department of Orthopaedic Surgery, Graduate School of Medicine, Gunma University, Maebashi-shi, Gunma 371-8511 Japan

**Keywords:** C-SWE, *Q*-index, SWDI, Screen score, Muscle monitoring

## Abstract

**Purpose:**

Continuous shear wave elastography (C-SWE) can be expected to be applied to portable muscle elasticity diagnosis. To establish diagnostic technology, it will be necessary to improve measurement techniques and quantitative measurement accuracy.

**Methods:**

In this study, we investigated two screen scores: the quality index (*Q*-index), which determines whether the intensity of a power Doppler image is appropriate, and the shear wave propagation direction index (SWDI), which determines the uniformity of shear wave propagation.

**Results:**

First, we performed numerical simulations with white noise and found that the coefficient of variation of shear wave velocity estimation was less than 5% when the normalized *Q*-index was greater than 0.27. Furthermore, regarding the SWDI, we clarified the relationship between the standard deviation in shear wave propagation direction and the SWDI. Next, the relationship between the *Q*-index and coefficient of variation of estimated shear wave velocity was evaluated through experiments using a tissue-mimicking phantom. The results showed that there was a negative correlation between the *Q*-index and the coefficient of variation, and the fluctuation of the propagation velocity could be inferred from the *Q*-index. Finally, we showed the results of applying the screen scores to muscle relaxation monitoring and confirmed its usefulness in clinical applications.

**Conclusion:**

By applying the screen scores, we showed improved stability in speed estimation in C-SWE, and demonstrated the possibility of clinical applicability.

**Supplementary Information:**

The online version contains supplementary material available at 10.1007/s10396-024-01439-7.

## Introduction

In orthopedics, it is necessary to evaluate the elastic dynamics of muscles, such as muscle expansion and contraction, and changes in tissue elasticity before and after exercise, rehabilitation, and treatment. Elastography has been researched and developed as an ultrasound diagnostic technique to noninvasively obtain viscoelastic information of living tissue [1, 2]. In 2016, liver elastography was approved for insurance coverage. Conventional shear wave elastography (SWE) uses acoustic radiation force to excite shear waves in human tissues, and estimates viscoelastic parameters from their propagation characteristics [3–6]. Studies have been conducted on muscle elasticity using SWE [7–9]. However, since ultrasound waves with large acoustic intensity are used to generate shear waves, it has been pointed out that there is a risk of temperature rise if hard tissues such as bones are near the region of interest (ROI) [10], or thermal and mechanical damage to cells [11, 12]. Therefore, careful consideration is required when using SWE in the orthopedic field. Furthermore, since parallel calculation processing is required for reconstruction of shear waves, ultrasound devices tend to be large and expensive. Therefore, it is difficult to use it in daily outpatient care, at the bedside, or in the field, such as at sporting events.

**Fig. 1:**
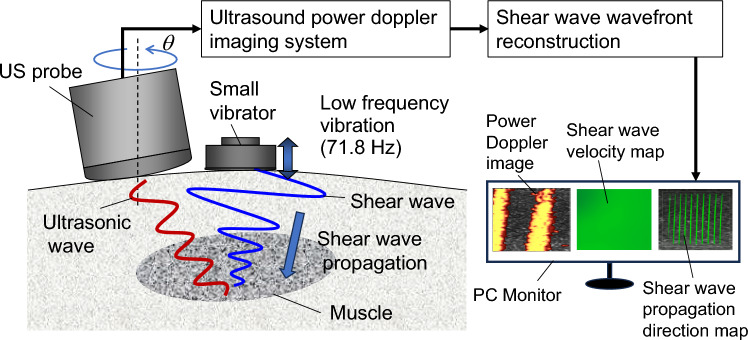
Schematic diagram of C-SWE

To solve these issues, methods for estimating shear wave velocity using mechanical excitation have been proposed [13–15]. Among them, continuous shear wave elastography (C-SWE) has been developed by Yamakoshi et al. [15, 16]. The C-SWE measurement system shown in Fig. 1 images the wavefront and velocity of shear waves, which are generated by a small vibrator (height and width are less than several centimeters). C-SWE allows color Doppler images to be used for image processing without modifying conventional ultrasound equipment with color Doppler functions, so that we can quantitatively obtain images of the hardness of living tissues by simply importing the color Doppler images to a PC for image processing. For this reason, although an ultrasound device is required, the imaging device is inexpensive and can be implemented not only in a stationary echo device but also in a tablet echo device. Therefore, it can be used not only in the medical room but also at the bedside, physical therapy room, and sporting events. In addition, C-SWE is characterized by the fact that the propagation of shear waves can be directly observed in real time, and because it does not use acoustic radiation pressure to produce shear waves, it is highly safe for living tissues.

To date, we have applied C-SWE to healthy subjects to measure the propagation velocity of shear waves for the biceps brachii, carpi radialis, semitendinosus, biceps femoris, gastrocnemius medialis, and tibialis anterior. Evaluation of intra- and inter-rater reliability showed that it was reliable as a measurement method [16, 17]. In addition, the propagation velocity of shear waves was measured using C-SWE while the tensile stress was varied on the rectus femoris muscle extracted from a donated body. We found that the relationship between tensile stress and elastic modulus was almost linear, suggesting that it is suitable for measuring passive stress in muscle [18].

However, it is necessary to improve measurement techniques and accuracy (quantitativity) to deal with dynamic changes in muscle elasticity (e.g., changes due to stretching and load such as exercise), which are important in the evaluation of locomotor function. For example, regarding the measurement position, it is necessary to understand the origin/insertion of the muscle on the B-mode image, and to confirm that the excitation point is placed on the extension line of the target muscle fibers. It is also desirable that the amplitude of the Doppler signal be sufficient for shear wave detection and that shear waves propagate uniformly in the same direction as the muscle fibers. In general, shear wave measurement has the problem that there is no indicator for operators to understand the measurement accuracy of shear waves, and it is difficult to distinguish between measurement variations and changes in elastic properties.

In this paper, we considered the application of screen scores to evaluate the measurement quality of acquired shear wave images to improve the measurement quality of C-SWE. An overview of C-SWE is provided in Sect. “Overview of C-SWE”. Section “Method: proposal for introducing screen scores” explains two types of screen scores. In Sect. “Results: numerical simulation and experimentation”, we conducted numerical verification as well as experimental verification. Finally, we applied the screen scores to muscle relaxation monitoring using C-SWE.

## Overview of C-SWE

In this section, we explain the principle of C-SWE [15] and the quality of reconstructed shear wave records.

### Principle of C-SWE

#### Reconstruction of shear wave wavefront

As shown in Fig. 1, a small vibrator is placed on the surface of the human body and continuously vibrates at low frequency. The excitation frequency, i.e., shear wave frequency, is ${f}_{{\mathrm{b}}}$ (71.8 Hz). The continuous shear wave is produced by the vibrator and propagates in muscle. Then the ultrasonic echoes are acquired with the ultrasound scanner to detect shear waves. Doppler signals are obtained from quadrature detector output. Under the conditions of Eq. (1), the wavefront of shear waves appears as a binary pattern that consists of zero and non-zero amplitudes of the Doppler signal on the power Doppler image (PD image) on the PC monitor.

1 $$f_{{\mathrm{b}}} = \frac{1}{2}\left( {m + \frac{1}{2}} \right)f_{{{\mathrm{PRF}}}} .$$

Here, $m$ is zero or an integer and ${f}_{PRF}$ is PRF of the ultrasound scanner.

#### Reconstruction of shear wave maps

PD images are acquired as motion pictures to reconstruct quantitative shear wave maps. Here, the quality of the PD image is affected by noise, which is caused by inherent tissue movements, clutter, and electrical circuit. Therefore, to suppress noise, two-dimensional Fourier transform is applied to PD images in the reconstruction process.

Applying two-dimensional Fourier transform to PD images, a raw shear wave complex amplitude map, which has amplitude and phase components, is obtained. After Fourier transformation of the phase component of the raw shear wave complex amplitude map, we obtain the reconstructed shear wave phase map, ${\theta }_{{\mathrm{CFI}}}$. After performing two-dimensional filter processing (Wiener filter, directional filter, low-pass filter (LPF)) on ${\theta }_{{\mathrm{CFI}}}$, a wave number map is calculated via spatial differentiation of ${\theta }_{{\mathrm{CFI}}}$. From the wave number map, we calculate a shear wave velocity map, ${v}_{\mathrm{est}}$, and a shear wave propagation direction map, ${\theta }_{\mathrm{prop}}$. Here, the shear wave propagation direction map displays the wavefront of shear waves. The shear wave velocity map and the shear wave propagation direction map are displayed as reconstructed quantitative shear wave maps on the PC monitor (Fig. 1). In this study, the shear wave velocity is assumed to be constant, and shear waves are assumed to propagate uniformly in the ROI.

### Examples of reconstructed shear waves

Excluding images that are unsuitable for shear wave velocity measurement is essential for improving the quantitative nature of shear wave velocity measurement. Here, we show examples of shear waves recorded using C-SWE. The experimental equipment was the same as that used in the experiment in Sect. “Experiment with a tissue-mimicking phantom to verify the effect of Q-index”. In the system used in this study, shear waves are displayed as propagating from right to left on the PC monitor.

#### Example 1

Movies 1a, b show examples of the PD image records of the biceps brachii of a healthy male. Shear waves propagate from right to left in the movies. The records were obtained with the subject sitting on a chair and his right forearm placed on a desk. Then PD images of the biceps brachii of the subject were recorded to PC. Depending on the measurement situation, there is a recording example (Movie 1a) that is considered to have high-quality shear waves, and a recording example (Movie 1b) that is considered to be of low quality. We intentionally created a condition in which vibrations were not well transmitted to the muscles to obtain Movie 1b. In recording examples that are considered to be of high quality, shear waves propagate uniformly from right to left. In addition, the wavefront propagates in a group, and the signal amplitude of the PD image is high. On the other hand, as shown in Movie 1b, in recording examples that are considered to be of low quality, the propagation direction of the shear wave wavefront is not uniform. In addition, the PD signal amplitude is low and scattered in the PD image in Movie 1b.

There are several possible reasons for the low-quality image in Movie 1b. First, one case is considered in which the propagation velocity differs for each muscle fascicle in the ROI. In this case, when a shear wave obliquely enters an interface with different velocities, the shear wave may be refracted or reflected. Furthermore, if there is bone outside the ROI, reflected shear waves may be visualized in the ROI. Therefore, when we assume that the propagation velocity of shear waves is constant and that shear waves uniformly propagate in the ROI, the shear waves shown in Movie 1b are not suitable for measurement. Therefore, in this case, the waves in Movie 1b should be removed.

In addition to the above case, let us consider other factors that affect the accuracy of shear wave velocity estimation. First, a decrease in the signal amplitude of a PD image is inappropriate because it affects shear wave velocity estimation. There are several possible reasons for the decrease in the signal amplitude of the PD image. If the contact between the ultrasound probe or vibrator and the skin surface is poor, shear waves will not be excited, or even if shear waves are excited, the resulting PD image would have a weak signal. If there is bone between the vibrator or ultrasound probe and muscle, and shear waves pass through the bone, the signal amplitude of the PD image will also be reduced.

#### Example 2

Other examples of reconstructed shear wave propagation direction records for the vastus lateralis muscle of a healthy male are shown in Movies 2a, b. The subject sat on a chair and PD images of the vastus lateralis muscle of the subject were recorded to PC. Then the reconstructed shear wave propagation direction records were calculated using C-SWE.

In muscle fibers, the propagation speed of shear waves generally differs between the fiber direction and the direction perpendicular to the fiber direction. Therefore, when measuring the propagation velocity in the fiber direction, it is desirable that shear waves uniformly propagate in the muscle fibers as plane waves. However, if there is rotation in the wavefront or if the wavefront does not propagate uniformly in the muscle fiber direction, the shear waves are regarded as not being suitable for measuring propagation velocity. In this study, when the muscle fiber direction and the shear wave propagation direction were within approximately 20°, they were considered to be horizontal to each other. Under this condition, the measured speed is expected to have a measurement velocity error equivalent to ${\mathrm{cos}}$(20) (= 0.94) compared to the ideal case where they are completely parallel.

Movie 2(a) is considered to be a desirable state in which shear waves propagate uniformly in the muscle fiber direction almost as a plane wave. On the other hand, Movie 2(b) is considered to be a case where the wavefront of shear waves has a vertical curvature and distortion occurs in the plane wave.

## Method: proposal for introducing screen scores

In this study, we apply two screen scores, the quality index (*Q*-index) and the shear wave propagation direction index (SWDI), which are independent from each other, to evaluate shear wave quality and improve the quantitativity of shear wave velocity measurement. The *Q*-index is a score indicating the strength of the signal amplitude of PD images. The SWDI is a score that evaluates whether the wavefront propagates uniformly within the imaging plane.

### *Q*-index

The displacement *ξ* due to shear waves is written as

2 $$\xi = \xi_{0} \sin \left( {\omega_{{\mathrm{b}}} t + \varphi_{{\mathrm{b}}} } \right).$$

Here, $\xi_{0}$ is the displacement amplitude of the shear wave, $\omega_{b}$ is the excitation angular frequency of the shear wave $({ } = 2{\uppi }f_{{\mathrm{b}}}$), and $\varphi_{b}$ is the phase of the shear wave. A shear wave receiving ultrasound signal $S\left( t \right)$ is expressed by Eq. (3).

3 $$S\left( t \right) = S_{0} {\mathrm{exp}}\left\{ {j\left( {\omega_{0} t + m_{{\mathrm{f}}} \sin \left( {\omega_{b} t + \varphi_{b} } \right) + \varphi } \right)} \right\}$$

Here, $S_{0}$ is the reception amplitude of ultrasound, $\omega_{0}$ is the angular frequency of ultrasound, $m_{{\mathrm{f}}}$ is the modulation coefficient $\left( {m_{{\mathrm{f}}} = \frac{{4\pi f_{0} \xi_{0} }}{c}} \right)$, *c* is the longitudinal wave velocity of human tissue, and $\varphi$ is the initial phase of ultrasound.

The Doppler signal obtained by orthogonally detecting the received ultrasound signal is expressed by Eq. (4).

4 $$\begin{aligned} I\left( t \right) & = G{\mathrm{cos}}\left( {m_{{\mathrm{f}}} \sin \left( {\omega_{{\mathrm{b}}} t + \varphi_{{\mathrm{b}}} } \right)} \right) \\ Q\left( t \right) & = G{\mathrm{sin}}\left( {m_{{\mathrm{f}}} \sin \left( {\omega_{{\mathrm{b}}} t + \varphi_{{\mathrm{b}}} } \right)} \right). \\ \end{aligned}$$

Here, $G$ is the proportionality coefficient that includes the amplitude of the received ultrasound signal. At this time, the amplitude spectrum of $I\left( t \right)$ and $Q\left( t \right)$ obtained via spectrum analysis of the excitation frequency component of the Doppler signal is expressed by Eq. (5).

5 $$A_{1} = 2G\left| {J_{1} \left( {m_{{\mathrm{f}}} } \right)} \right|.$$

Here, $J_{1}$ is the first-order Bessel function. Therefore, the power spectrum of the excitation frequency of the Doppler signal is expressed by Eq. (6).

6 $$A_{1}^{2} = 4G^{2} \left( {J_{1} \left( {m_{{\mathrm{f}}} } \right)} \right)^{2} .$$

The first-order Bessel function can be approximated by Eq. (7) when $m_{f} \cong 0$.

7 $$J_{1} \left( {m_{f} } \right) = \frac{1}{2}m_{f}$$

Therefore, Eq. (6) becomes Eq. (8).

8 $$A_{1}^{2} = 4G^{2} \times \left( {\frac{1}{2}m_{{\mathrm{f}}} } \right)^{2} { } { } = G^{2} m_{{\mathrm{f}}}^{2} = G^{2} \left( {\frac{{4\pi f_{0} }}{c}} \right)^{2} \xi_{0}^{2}$$

In other words, the power of the Doppler signal represents the power of the displacement amplitude $\xi_{0}$. Therefore, the amplitude of the PD image can be used as an index representing the magnitude of the displacement amplitude in the shear wave image. Here, $A_{1}$ is defined as the *Q*-index.

 In Eq. (8), $G$ is a parameter determined depending on the internal configuration of the ultrasound diagnostic device, and it is difficult to obtain the power of the Doppler signal as an absolute physical quantity. For this reason, in practice, for example, a phantom experiment is performed by determining the excitation voltage for the vibrator that elicits shear waves, and the Doppler signal power is determined under conditions where the shear wave amplitude is considered to be sufficiently high within the ROI. By standardizing the Doppler signal power obtained with this value, it is necessary to eliminate the difference between ultrasound diagnostic apparatuses.

### SWDI

Next, we discuss the application of shear wave wavefront scores. Here, we devised the *SWDI*, an index that numerically expresses the deviation of the wavefront from a plane wave. In other words, the *SWDI* is an index for evaluating how much the direction of shear wave propagation varies from the average direction of propagation.

The *SDWI* is expressed by Eq. (9).

9 $${\mathrm{SWDI}} = 100 - \frac{1}{KS}\iint {\left| {\theta_{{{\mathrm{prop}}}} \left( {x,z} \right)} \right|^{p} {\mathrm{d}}x{\mathrm{d}}z}.$$

Here, ${\theta }_{\mathrm{prop}}\left(x,z\right)$ is the shear wave propagation direction map and $S$ is area of the evaluation ROI. *p* and *K* are parameters that set the sensitivity to the deviation of the SWDI propagation direction from the average propagation direction. *p* is a positive integer value. Also, if the SWDI value is 0 or less, the value is set to 0, and if the SWDI value is 100 or more, the value is set to 100.

## Results: numerical simulation and experimentation

We performed a numerical simulation of the *Q*-index and the SWDI. We also conducted an experiment using a tissue-mimicking phantom as well as a clinical study to verify the effects of applying the screen scores. We note that in the experiments, the shear wave velocity and the shear wave propagation direction maps were calculated after operating a directional filter [19, 20], a Wiener filter [21], and a LPF on the reconstructed shear wave phase map, as already described in Sect. “Reconstruction of shear wave maps”.

### Numerical simulation of *Q*-index and *SWDI*

#### Numerical simulation of *Q*-index

 We executed the numerical simulation and experiment to investigate the relationship between the *Q*-index and the coefficient of variation (CV). We performed a numerical simulation of reconstruction of shear waves via C-SWE. The simulation method is described in Ref. [22].

In the simulation, the ultrasound echo signal where the shear wave exists (Eq. (1) in Ref. [22]) was considered. Here, the shear wave was modeled as a sinusoidal wave (Eq. (5) in Ref. [22]). The real part, $Im$, and the imaginary part, $Re$, of quadrature detection output are expressed by Eqs. (10) and (11), respectively.

10 $${\mathrm{Im}} = y_{0} {\mathrm{cos}}\left( {m_{f} {\mathrm{sin}}\left( {\omega_{b} t + {\boldsymbol{kx}} + \varphi_{b} } \right)} \right) + a\_n$$

11 $${\mathrm{Re}} = y_{0} {\mathrm{sin}}\left( {m_{f} {\mathrm{sin}}\left( {\omega_{b} t + {\boldsymbol{kx}} + \varphi_{b} } \right)} \right) + a\_n.$$

Here, ${y}_{0}$ is the amplitude of the Doppler signal, ${\boldsymbol{k}}$ is the wave number vector of the shear wave, ${\boldsymbol{x}}$ is the position vector, and $a\_n$ is additive noise. The quadrature detection output was the Doppler signal to be simulated. In addition, noise was added to the Doppler signal to investigate the relationship between the *Q*-index and the CV.

The simulation code was written in C project using Visual Studio 2022 (Microsoft) in this study. Spatial sizes of the simulation in the *x* and *z* directions were 25.6 and 22.1 mm, respectively. The pixel sizes in the *x* and *z* directions were 64 and 145 pixels, respectively. The frequencies of ultrasound and shear waves were 6.8 MHz and 71.8 Hz, respectively. The longitudinal wave velocity was 1500 m/s. The amplitude of the shear wave and shear wave velocity was 10 µm and 2.0 m/s, respectively. The amplitude of the Doppler signal was 1.0. The attenuation coefficient of the shear wave in the *z* direction was set to 0.005 dB/pixel (i.e., a total of 0.73-dB attenuation in the depth direction of the ROI, 22.1 mm). The parameters (amplitude, velocity, and attenuation coefficient) according to the shear wave were determined from the experiments in our group. In the case of noise addition, Gaussian white noise component with the signal amplitude of 1.0 was added to Doppler signals. In total, 16 packets of Doppler signals were obtained, where the time step was 0.00116063 s.

The raw shear wave phase map was obtained by calculating the angle of the packets of Doppler signals. Then two-dimensional Fourier transform was applied to the raw shear wave phase map, and the reconstructed shear wave map was calculated [15]. The simulation yielded “the shear wave velocity map,” “the shear wave phase map,” and “the shear wave propagation direction map.”

Here, Gaussian white noise was generated as additive noise using the rand function that generates pseudorandom numbers. The standard deviation (SD) of additive noise was set from 0 (no noise) to 3. For each SD, calculation was executed 10 times where the initial conditions of a random seed were constant. In the simulation, the quality of shear waves in the ROI was proportional to the variance of the noise amplitude. Therefore, the averaged velocity in the ROI was calculated for evaluation.

**Fig. 2:**
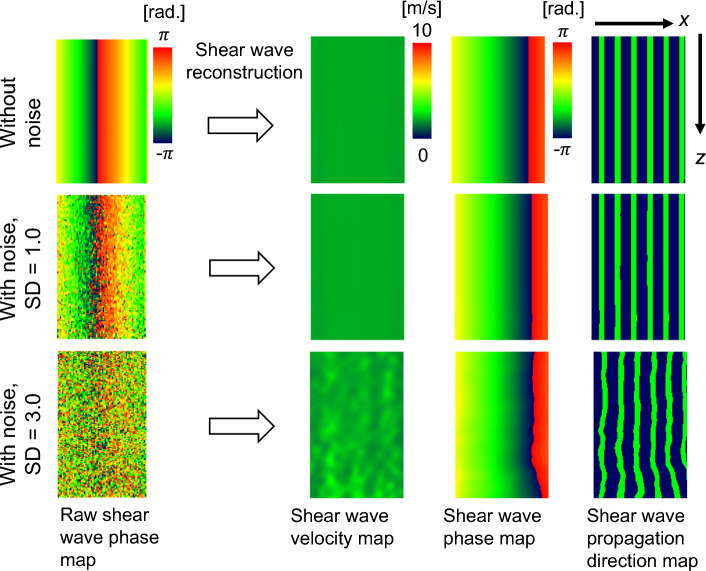
Numerical simulation results of raw shear wave phase, shear wave velocity, shear wave phase, and shear wave propagation direction maps with and without white noise

**Fig. 3:**
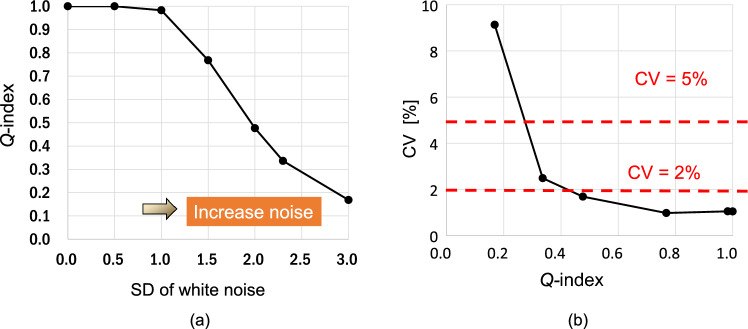
Numerical simulation results. **a** *Q*-index vs. standard deviation of white noise. **b** Coefficient of variation of estimated shear wave propagation velocity vs. *Q*-index. *Q*-index results are averaged values within the ROI and normalized by the index without white noise

Figure 2 shows the simulation results obtained suing C-SWE for three cases, i.e., when there is no noise and the SDs of the additive noise are 1.0 and 3.0. When there is no noise, the shear wave velocity map has the same value within the ROI, and the shear wave propagation map shows that shear waves propagate uniformly as plane waves. On the other hand, when additive noise is added, the velocity values vary and the wavefronts of shear waves are not uniform. Figure 3a shows the simulation results of the *Q*-index when the SD of noise is changed. The results show that as the SV of the noise increases, the *Q*-index decreases. Figure 3b shows the relationship between the *Q*-index and the CV of the estimated propagation velocity. From the result, for example, to generate a CV of 5% or less, the *Q*-index needs to be 0.27 or more, while to generate a CV of 2% or less, the *Q*-index needs to be 0.42 or more. Note that the *Q*-index in Fig. 3a, b is normalized by the value of the *Q*-index without noise.

From the simulation results, we confirmed that measurement accuracy decreased when the value of the *Q*-index decreased. We believe that this trend would be reproduced in actual measurements. Please note that the quantitative relationship between the threshold of the *Q*-index and the CV in actual measurements would be different from the simulation results. The value would be varied due to the measurement environment and the measurement site. Therefore, we need to carefully determine the threshold in a preliminary test.

#### Numerical simulation of SWDI

**Fig. 4:**
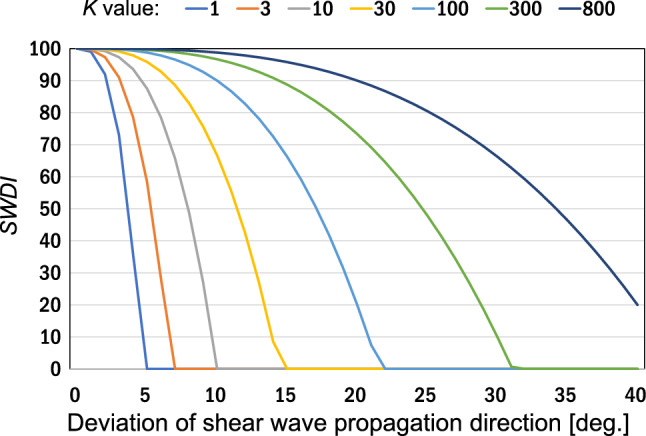
Numerical simulation results: *SWDI* vs. deviation of shear wave propagation direction with different *K* values. Constant value of *p* (= 3) is used

We calculated Eq. (9) by changing the value of ${\theta }_{\mathrm{prop}}\left(x,z\right)$ from 0° to 40°. Figure 4 shows the relationship between shear wave propagation direction deviation and the *SWDI* value when *p* = 3. The smaller the value of *K*, the higher the sensitivity of changes in the *SWDI* value to deviations in the propagation direction. In addition, for example, when *p* = 3 and *K* = 10, if SWDI > 90 is selected, the direction deviation will be less than 5°. When the wavefront is considered to be a plane wave, as in Movie 2(a), the SWDI is as high as 99.7, and when there is a deviation in the propagation direction, as in Movie 2(b), the SWDI becomes 67.2.

From the result, we expect that by determining the threshold of the SWDI, shear waves propagating in the same direction will be extracted. As in the case of the *Q*-index, the quantitative relationship between the threshold of the SWDI and the shear wave propagation direction in actual measurements would be different from the simulation results. Therefore, we need to carefully determine the threshold in a preliminary test.

### Experiment with a tissue-mimicking phantom to verify the effect of *Q*-index

In Sect. “Numerical simulation of Q-index”, the values of the *Q*-index were decreased with noise addition. In the experiment, we decreased the *Q*-index by reducing the intensity of a vibrator.

The relationship between the *Q*-index and the CV through experiments using tissue-mimicking phantoms was evaluated. An elastic phantom (sodium alginate, calcium sulfate, trisodium phosphate 12-hydrate, glycerol, and water, 200 ml) [23] was used as a tissue-mimicking phantom. A linear array probe (9L-D, GE HealthCare) and an ultrasound scanner (LogiqS8, GE HealthCare) were used. Power Doppler imaging (PDI) mode was executed to obtain PD images. The frame rate and the ultrasound frequency of PDI mode were 29 fps and 4.2 MHz, respectively. The excitation frequency of a vibrator was set to 71.8 Hz, at which a shear wave wavefront image appears on the PD image. After positioning the probe and the vibrator, they were fixed with a jig. The sizes of the ROI were 22.1 and 26.5 mm in the depth and horizontal directions, respectively. The system visualizes shear waves at 5.54 s/frame. PD images used to estimate the shear wave velocity were 16 packets and stored to PC for calculation. The pixel sizes of PD images were 145 and 224 pixels in the depth and horizontal directions, respectively. Two-dimensional Fourier transform was performed on PD images to obtain the raw shear wave complex amplitude map. Then the reconstructed shear wave phase map was calculated. After a LPF, a directional filter and a Wiener filter were applied to the reconstructed shear wave phase map, “the shear wave velocity map” and “the shear wave propagation direction map” were outputted for each frame. Here, the LPF was applied to remove the high-frequency noise of the spectrum of the reconstructed shear wave phase map. The directional filter was applied to extract shear waves that propagate in the -*x* direction. The sizes applied to the Wiener filter were determined as a constant multiple of the peak amplitude value of the spectrum of the reconstructed shear wave phase map.

**Fig. 5:**
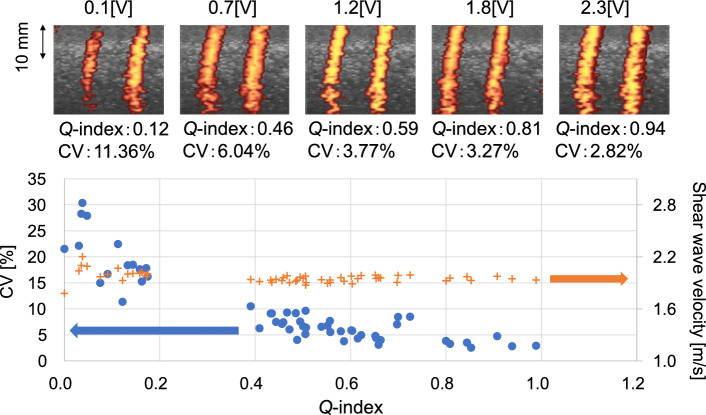
Experimental results of tissue-mimicking phantom. Changes in shear wave velocity, CV, and *Q*-index with different applied voltage

To intentionally reduce the *Q*-index, the voltage of the vibrator was set to the maximum value of 2.3 V as an initial value, and then the voltage was gradually decreased. The measurement was performed until the wavefront of the shear wave disappeared from the PD image. Figure 5 shows the calculation results of the *Q*-index and the CV of the estimated propagation velocity when changing the voltage. This tendency agrees with the simulation results in Fig. 3b. It was found that there was a negative correlation between the *Q*-index and the CV, and the fluctuation of the propagation velocity at that time could be inferred from the *Q*-index. The estimated velocities of shear waves were 1.95 ± 0.02 m/s at *Q*-index > 0.7 and 1.96 ± 0.07 m/s at *Q*-index < 0.7.

### Clinical study for muscle monitoring

#### Preliminary in vivo experiment

**Fig. 6:**
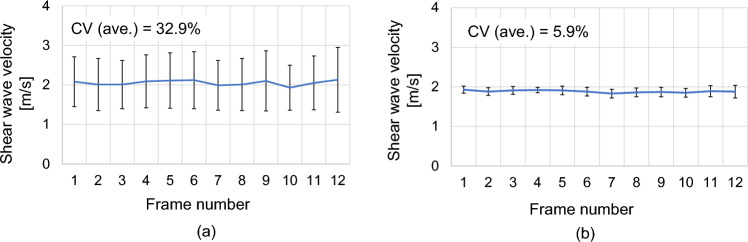
Results of in vivo experiments. **a** Shear wave velocity was estimated with all the pixels in the ROI. **b** Shear wave velocity was estimated with the pixels of high *Q*-index

Before investigating muscle relaxation monitoring, we executed a preliminary in vivo experiment. The shear wave velocity was estimated for the vastus lateralis muscle of a healthy male. The subject sat on a chair, and PD images of the vastus lateralis muscle of the subject were stored to PC. The velocity of shear waves was calculated from the stored data. Since the human tissue structure is more complex than a phantom, we applied the screen score of the *Q*-index for each pixel of the ROI in the in vivo study. In other words, only the high-quality pixels were used for the velocity estimation. Figure 6a shows the results of the estimated shear wave velocity when all the pixels in the ROI were used. Figure 6b shows the results where pixels with the *Q*-index (> 0.7) were used. The results indicate that we improved the CV from 32.9 to 5.9% by applying the *Q*-index to each pixel. The estimated velocities of shear waves were 2.05 ± 0.06 m/s and 1.88 ± 0.03 m/s for the results in Fig. 6a, b, respectively.

#### Muscle relaxation monitoring

An example of application of the screen scores to muscle relaxation monitoring is presented here. Skeletal muscle tension is not only controlled by the upper central nervous system, spinal cord, and peripheral nerves but is also influenced by various factors, including autonomic nerves, psychological conditions, pain, and external stimuli (e.g., auditory, visual, tactile). Therefore, muscle elasticity changes every moment. In clinical experiments, it is necessary to have a situation where the influence of external stimulation is sufficiently small, the muscle elasticity of skeletal muscle changes uniformly through intervention, and muscle tension can be measured using other methods.

An experiment was conducted under conditions in which a muscle relaxant was used during general anesthesia. Elastic changes were measured using C-SWE while objectively evaluating muscle tone using muscle relaxation monitoring. A muscle relaxation module (AF-201P, Nihon Kohden) was used for muscle relaxation monitoring. In the experiment, we investigated the relationship between the degree of muscle relaxation and elasticity (i.e., velocity) before and after applying the screen scores.

**Fig. 7:**
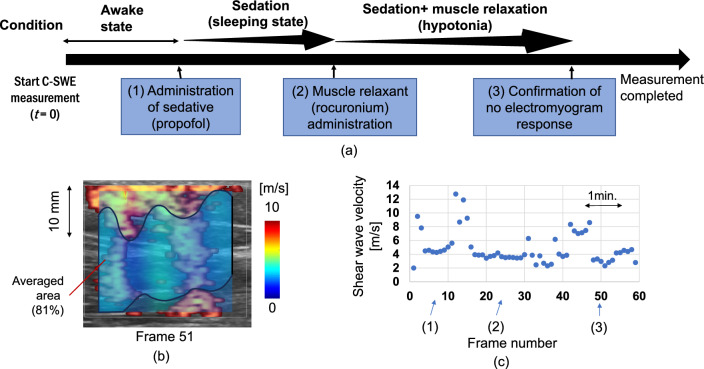
**a** Flow in muscle monitoring experiment. **b** Reconstructed shear wave velocity map and calculation area. **c** Estimated shear wave velocity vs. frame number before applying screen scores

Here, an example of one patient who was scheduled to undergo surgery under general anesthesia at an orthopedic clinic is presented. The experimental condition of the ultrasound scanner was the same as that described in Sect. “Experiment with a tissue-mimicking phantom to verify the effect of Q-index”. The measurement site was the vastus lateralis muscle. During the experiment, the patient laid down on a bed. A vibrator and the ultrasound probe were put on the muscle. The position of the probe was determined so that the muscle fiber direction and shear wave propagation direction were the same. Figure 7a shows the measurement procedure. *t* = 0 (Frame #0) is the time when the C-SWE measurement started. The timing of the C-SWE visualization is 5.54 s/frame. A sedative (propofol) was administered in the awake state to create an anesthetized state (Frame #7). A muscle relaxant (rocuronium) was administered under sedation (in a sleeping state) (Frame #23). At the 50th frame, zero electromyogram response (strong muscle relaxation) was confirmed. The pixels larger than *Q*-index > 0.7 were used for the shear wave velocity estimation. Figure 7b shows the shear wave velocity map and calculated area for Frame #51. Figure 7c shows the estimated shear wave velocity using C-SWE.

**Fig. 8:**
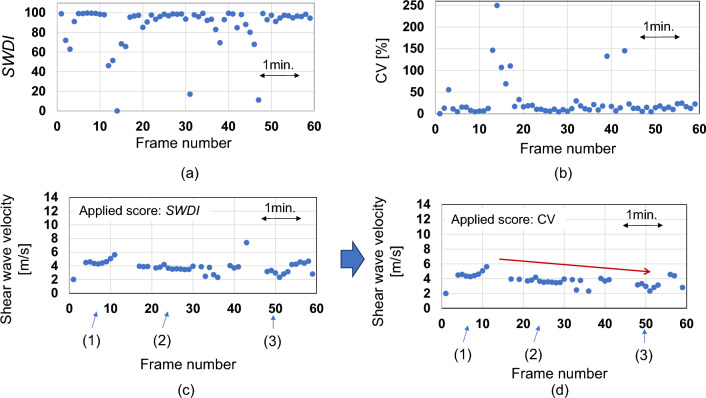
**a** *SWDI* and **b** CV vs. frame number in muscle monitoring experiment. **c**, **d** Estimated shear wave velocity vs. frame number after applying screen scores

To apply the screen scores, we considered the *SWDI* and the CV shown in Fig. 8a, b, respectively. *Q*-index > 0.7 was chosen for high-quality images based on the results of the phantom experiments in Sect. “Experiment with a tissue-mimicking phantom to verify the effect of Q-index”, where CV < 10% and the standard deviation was 0.02 m/s at *Q*-index > 0.7. SWDI > 90 was chosen by checking shear wave records on the PC monitor. The value was determined so that by applying SWDI > 90, the shear propagation angle from the vertical direction was approximately less than 20° in the shear wave propagation direction map. Movies 3(a), (b), and (c) and Movies 4(a), (b), and (c) show PD image records and shear wave records of Frame #5, #25, and #51, respectively. These records were judged as high-quality images. On the other hand, the PD image record and shear wave record in Movies 3(d) and 4(d) were excluded due to the low SWDI, 67.8. The PD image record and shear wave record in Movies 3(e) and 4(e) were excluded due to the high CV, 22.5%. The *SWDI* of Frame #14 dropped to zero, which is due to the small amplitude of the Doppler signal. Figure 8c shows the shear wave velocity after applying the SWDI screen score, where the images with *SWDI* > 90 were chosen as high-quality images. Figure 8d shows the shear wave velocity after applying the CV screen score, where the images with CV < 20 were chosen as high-quality images.

Shear wave propagation abnormalities mainly due to the subject’s body movements were automatically identified using the screen scores and excluded as data unsuitable for evaluation. After excluding specific data, a gradual decrease in muscle elasticity was observed. From the results, C-SWE incorporating the screen scores was effective as an indicator for determining muscle relaxation effects. The calculation areas of Frame #5, #25, #51, #46, and #43 were 95, 81, 70, 89, and 77%, respectively. In the shear wave propagation direction maps in Movie 4, only the calculation areas (i.e., *Q*-index > 0.7) are visible, and non-calculated areas were processed to be transparent. The velocity variations at Frame #1 to #6 (i.e., before the sedative was administered) were 2.00–9.50 and 2.00–4.58 m/s before and after applying the screen scores, respectively.

## Discussion

By applying the screen scores, we showed improved stability in shear wave velocity estimation using C-SWE, and also demonstrated the possibility of clinical applicability. However, there are issues that were found in the experiments and issues associated with the C-SWE measurement environment.

### Effect of body movement

 Compared to measurements performed using the tissue-mimicking phantom, fluctuations and outliers in estimated velocity values were noticeable in the clinical study. One of the reasons is thought to be the body movements of the subject and the person performing the measurement. To reduce the influence of measurement errors due to body movements, it is desirable to improve real-time performance compared to human movements and pulses (about 60 Hz). The system used in this study visualizes shear waves at 5.54 s/frame, and PD images used to estimate the shear wave velocity consisted of 16 packets. Real-time performance can be improved by reducing the number of packets of PD images used to calculate shear waves. For example, if four packets of PD images are used, the time required to collect data can be reduced to 1.39 s, making it possible to improve real-time performance. Such high-speed video playback can reduce the effects of disturbances such as body movements.

### Measurement area limit in depth direction

In the simulation in Sect. “Numerical simulation of Q-index”, we used the initial amplitude of the average shear wave obtained in our experiments, i.e., 10 µm. At this time, when the standard deviation of additive noise was 1.0, the CV of the estimated shear wave velocity was 1.1%.

Let us consider a case where the measurement target is located deeper than the muscle, such as the liver. For example, we assume that the shear wave attenuation constant is 4.3 dB/cm [24]. When the displacement at the skin surface is 100 µm, the displacement of shear waves decreases to 8.4, 3.1, and 0.7 µm at depths of 5, 7, and 10 cm, respectively. According to the simulation in Sect. “Numerical simulation of Q-index”, when the initial input value of shear waves decreases to 5, 2, and 1 µm, the CV increases to 1.4, 3.8, and 54.0%, respectively. Therefore, when the shear wave amplitude in the target region is small, it is considered necessary to make adjustments to keep the amount of displacement constant. For example, we would make adjustments by increasing the intensity of the vibrator or decreasing the frequency of shear waves.

### Placement angle of vibrator with respect to ultrasound probe

Considering measurement accuracy, it is desirable that the ultrasound probe and vibrator be in a straight line. In this study, the distances between the ultrasound probe and vibrator were about 2–3 cm, and thus the total length of the ultrasound probe and vibrator was about 10 cm. Therefore, we conducted the experiment on the vastus lateralis muscle since the skin surface has enough space so that measurement equipment can be sufficiently installed. However, in areas such as the trapezius muscle, the ultrasound probe and vibrator need to be placed at an angle rather than in a straight line.

As shown in Fig. 1, when the vibrator and ultrasound probe have an angle in the $\theta$ direction, correction should be made based on the relational expression $v{\prime}=v{\mathrm{cos}}\theta$ between the estimated velocity $v{\prime}$ using C-SWE and the true value $v$, which we want to measure. For example, when the shear wave velocity $v$ of the muscle fibers is 3 m/s, if $\theta$ is 5, 10, and 20°, the estimated $v{\prime}$ will be 2.98, 2.95, and 2.89 m/s, respectively. However, it is difficult to conduct experiments to reduce $\theta$ in narrow measurement areas with curvature such as the masseter muscle, and this requires miniaturization of the device and ingenuity in its arrangement depending on the measurement target.

As described above, in actual use of the screen scores, it is necessary to know in advance how to assign the appropriate score according to the body part to be measured, the excitation frequency and intensity, etc. Furthermore, since the value of the *Q*-index differs depending on the device, device-independent standardization is required. For standardization, a method such as using the *Q*-index value of a predefined phantom as a standard would be considered. Please note that the same ultrasound scanner was used throughout the experiments in this study, so we did not need standardization in this study.

### Applicability of simulation model and screen scores

In the simulation in this study, we mainly assume environmental noise (electrical noise generated from amplifiers, changes in conversion efficiency, thermal noise) generated in the ultrasound system as the fundamental noise consideration. Gaussian noise is widely used as the noise to evaluate ultrasound images such as B-mode images [25]. In addition, one of the keys of the C-SWE method is usage of conventional Doppler signals. Therefore, in this study, we investigated the relationship between the measurement quality and noise as a fundamental study to improve the measurement accuracy. We found the usefulness of the *Q*-index to exclude the unnecessary images or pixels, improving the CV. In addition, the *SWDI* also removed unnecessary shear wave records, which generated a larger CV, as shown in Fig. 8a, b. Although these indexes are qualitative, we believe the proposed concept is useful in the clinical setting and is an important first step toward future advanced studies.

However, in actual clinical practice, the measurement environment becomes more complex and the environment varies over time. Therefore, for example, we need to consider the characteristics of ultrasound scanners and probes to determine the optimum parameters. In addition, we need to consider tissue heterogeneity, tissue movements, clutter, and so forth. For tissue heterogeneity, speckle noise, reflection, refraction, or scattering should be considered. Furthermore, the model and the screen scores proposed in this study are qualitative. Tissue-mimicking phantoms and simulation models including noise closer to reality should be considered for further investigation to improve screen scores. Increasing in vivo experimental data and determining parameters using artificial intelligence would be a technical challenge.

## Conclusion

In this study, we proposed the application of screen scores to establish muscle elasticity diagnosis technology using C-SWE. It was found that by selecting data using the* Q*-index, the fluctuations in the estimated velocity values were reduced. In addition, by applying the screen scores to a muscle relaxation monitor, it became possible to more clearly understand the decline in muscle elasticity that accompanies the administration of muscle relaxants. Based on the present results, we confirmed that it is useful for diagnostic technology.

Currently, there is a need for a C-SWE system that automatically determines whether measured images are suitable for diagnosis. By applying the screen score system, we expect the development of an advanced system that extracts only high-quality images. It is also expected to be applied to a system that calculates screen scores from measured images and displays evaluation values on the screen in real time. This could also be used as an educational tool for practitioners.

Finally, there are some future challenges facing the proposed screen scores. It will be necessary to quantitatively relate simulations and phantom experiments regarding the *Q*-index and the SWDI. For example, the quantitative relationship between the *Q*-index and the CV with addition of noise should be investigated. In addition, the quality factors in this paper do not take into account heterogeneous structures such as the influence of fascia and reflections from bones. Therefore, it will be necessary to consider the heterogeneous structure. Furthermore, it will be necessary to consider a system that can determine optimal screen score parameters based on the excitation frequency and intensity. By establishing technology to comprehensively simulate these factors and introducing auto-calibration, we expect to improve the functionality of the screen scores proposed in this study.

## Supplementary Information

Below is the link to the electronic supplementary material.

Movie 1: Examples of shear wave records of biceps brachii. PD image records considered to be of (a) high and (b) low quality. Movie 2: Examples of shear wave propagation direction records of vastus lateralis muscle with (a) high and (b) low SWDI values. Movie 3: Results of shear wave records of vastus lateralis muscle in clinical study. PD image records of Frame #5, #25, and #51 are high-quality images. PD image records of Frame #46 have low SWDI. PD image records of Frame #43 of CV is larger than 20%. Images of Frame #43 and #46 are excluded. Movie 4: Results of shear wave records of vastus lateralis muscle in clinical study. Shear wave propagation direction records for high-quality images (Frame #5, #25, and #51) and excluded images (Frame #43 and #46)
