# Supplementary material for: Examination of rapid adjustment system based on screen score obtained using continuous shear wave elastography
Source: J Med Ultrason (2001). 2024 Apr 12;51(3):407–18. doi: 10.1007/s10396-024-01439-7 (PMC13046664; doi:10.1007/s10396-024-01439-7)
Supplement: Supplementary file 1 — Movie 1: Examples of shear wave records of biceps brachii. PD image records considered to be of (a) high and (b) low quality. Movie 2: Examples of shear wave propagation direction records of vastus lateralis muscle with (a) high and (b) low SWDI values. Movie 3: Results of shear wave records of vastus lateralis muscle in clinical study. PD image records of Frame #5, #25, and #51 are high-quality images. PD image records of Frame #46 have low SWDI. PD image records of Frame #43 of CV is larger than 20%. Images of Frame #43 and #46 are excluded. Movie 4: Results of shear wave records of vastus lateralis muscle in clinical study. Shear wave propagation direction records for high-quality images (Frame #5, #25, and #51) and excluded images (Frame #43 and #46) [file 10396_2024_1439_MOESM1_ESM.pptx]

## Slide 1
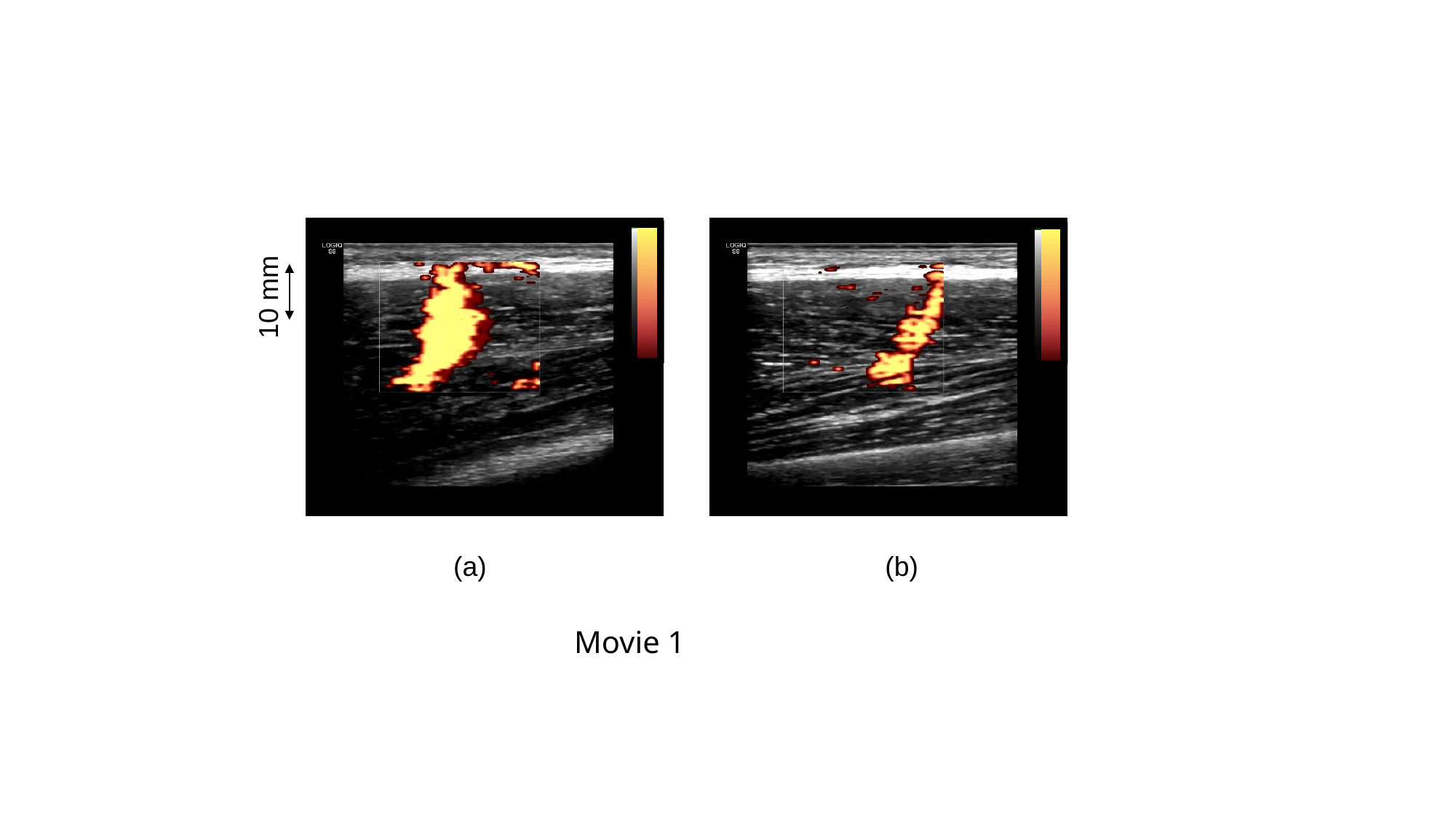

10 mm
(a)
(b)
Movie 1

## Slide 2
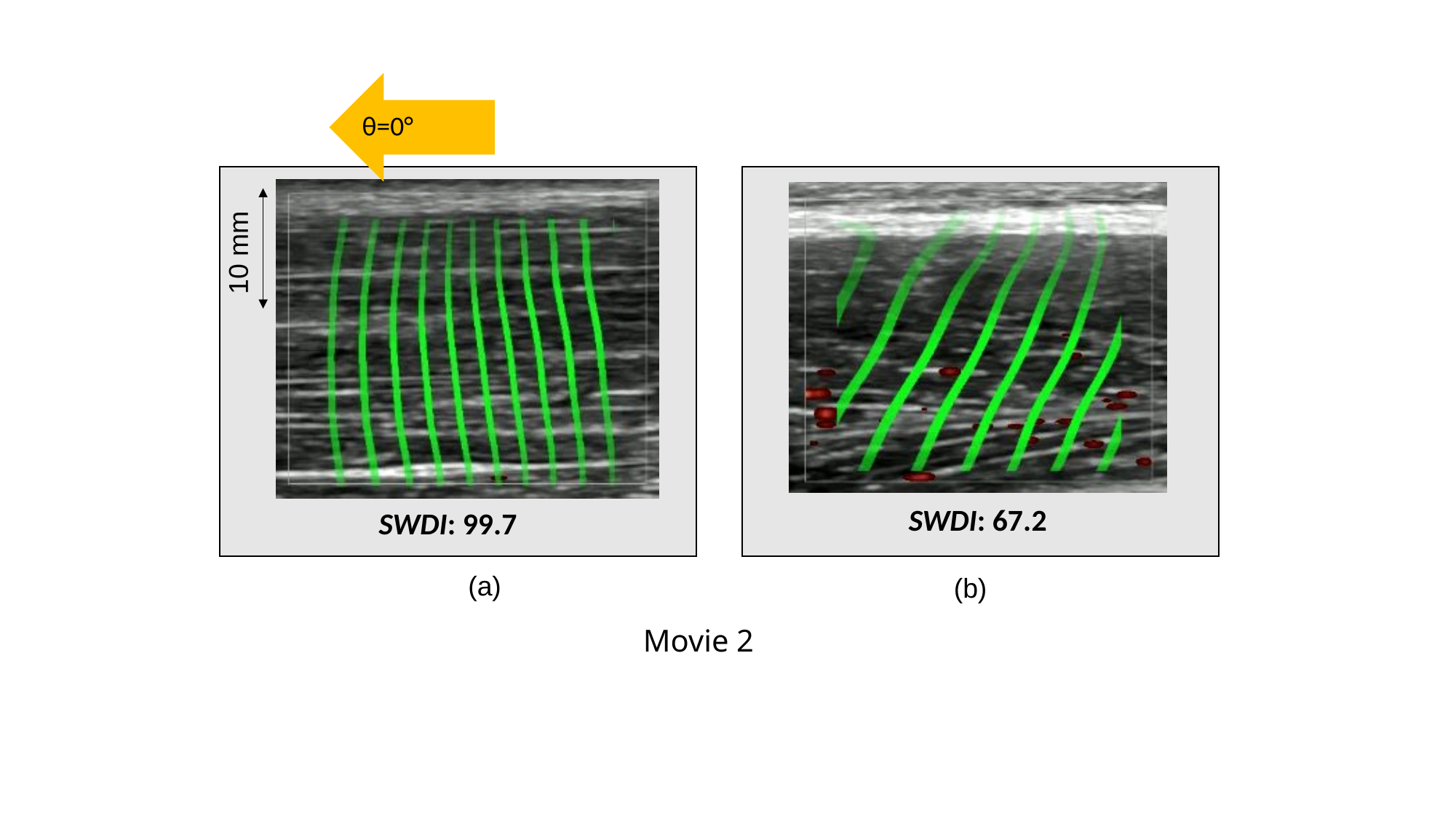

θ=0°
10 mm
SWDI: 67.2
SWDI: 99.7
(a)
(b)
Movie 2

## Slide 3
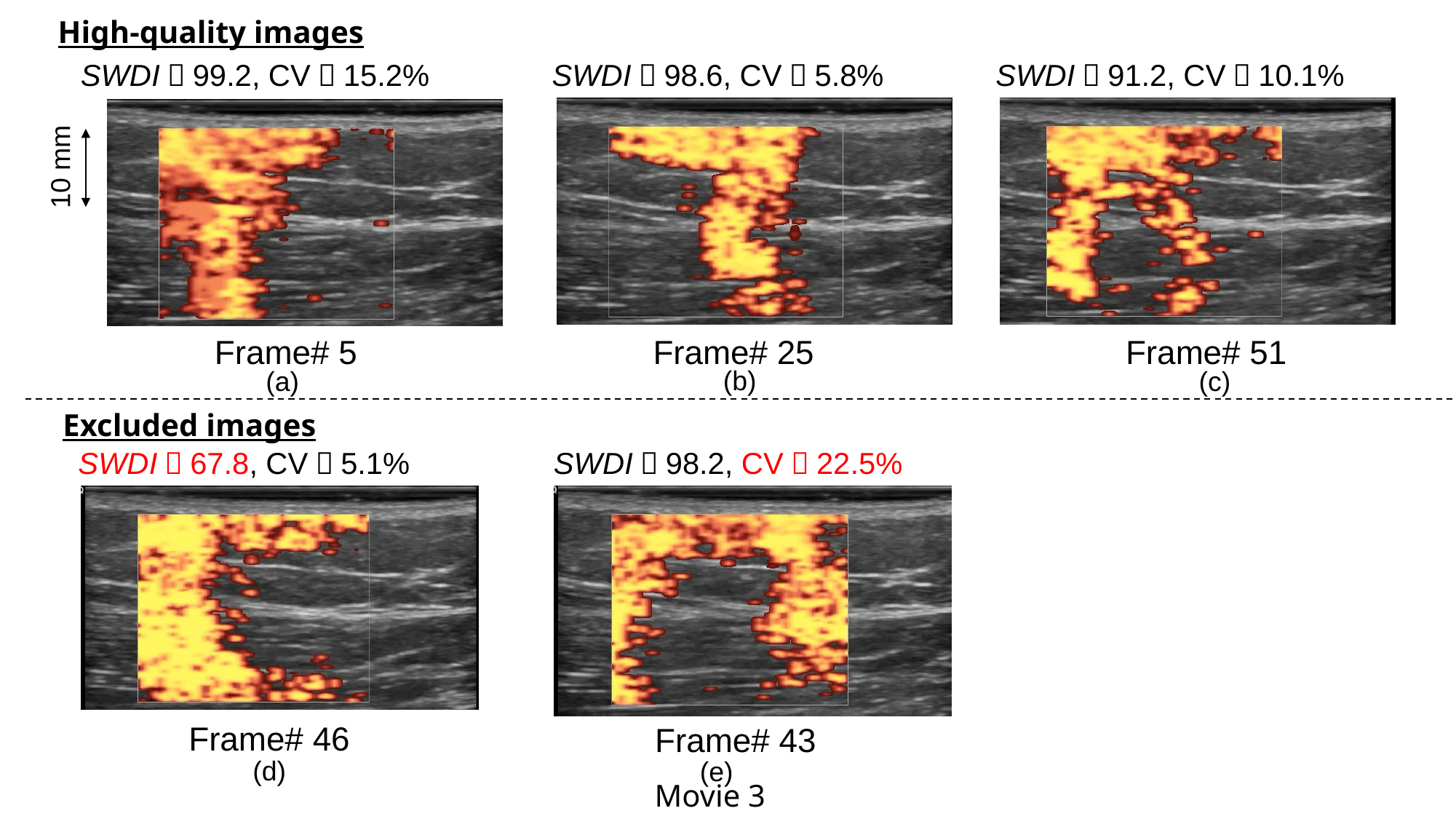

High-quality images
SWDI：99.2, CV：15.2%
SWDI：98.6, CV：5.8%
SWDI：91.2, CV：10.1%
10 mm
Frame# 5
Frame# 25
Frame# 51
(b)
(a)
(c)
Excluded images
SWDI：67.8, CV：5.1%
SWDI：98.2, CV：22.5%
Frame# 46
Frame# 43
(d)
(e)
Movie 3

## Slide 4
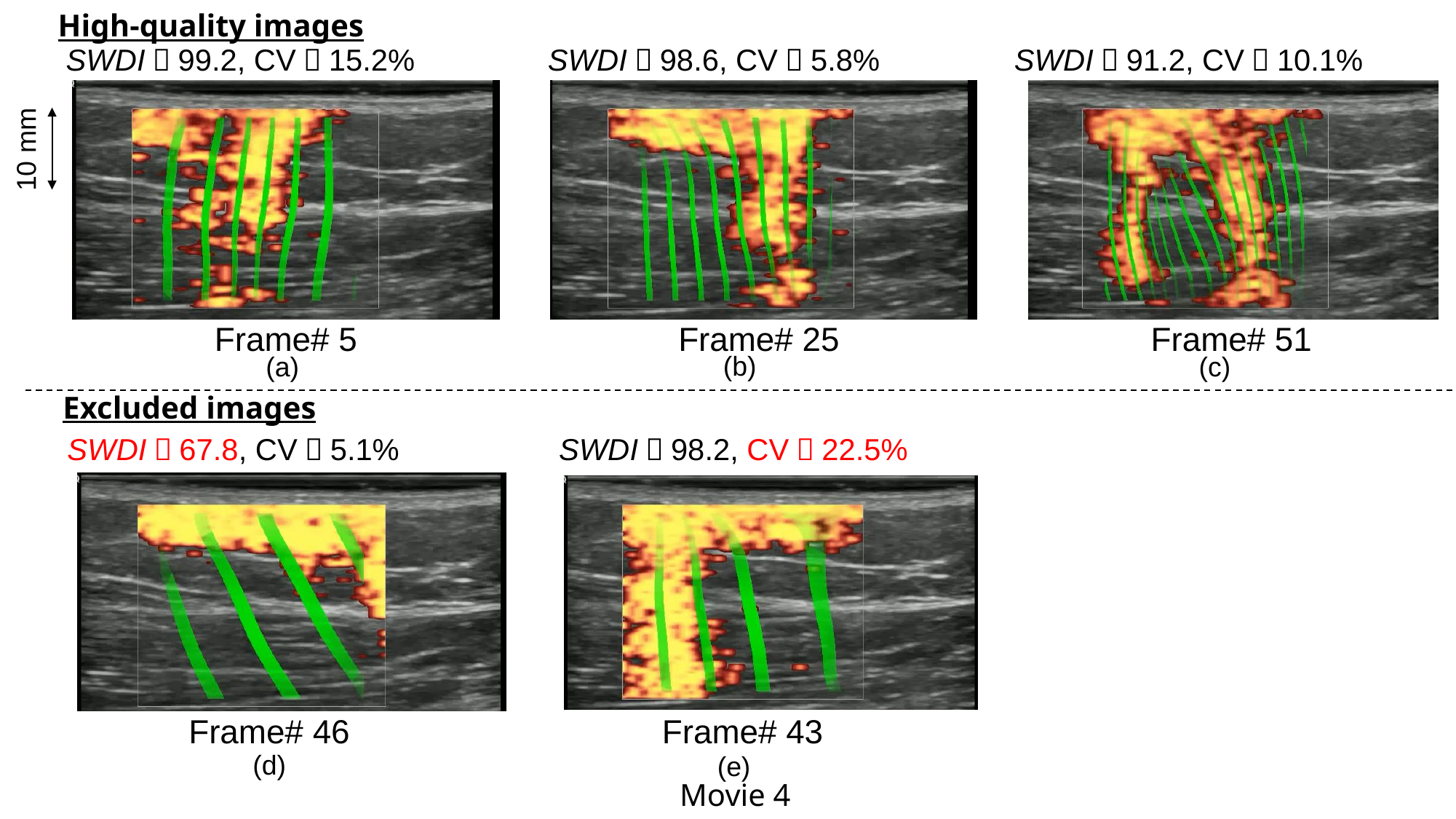

High-quality images
SWDI：99.2, CV：15.2%
SWDI：98.6, CV：5.8%
SWDI：91.2, CV：10.1%
10 mm
Frame# 5
Frame# 25
Frame# 51
(b)
(a)
(c)
Excluded images
SWDI：67.8, CV：5.1%
SWDI：98.2, CV：22.5%
Frame# 46
Frame# 43
(d)
(e)
Movie 4
